# Monitoring of Intracranial Pressure in Patients with Traumatic Brain Injury

**DOI:** 10.3389/fneur.2014.00121

**Published:** 2014-07-16

**Authors:** Christopher Hawthorne, Ian Piper

**Affiliations:** ^1^Clinical Lecturer, Academic Unit of Anaesthesia, Pain and Critical Care Medicine, University of Glasgow, Glasgow, UK; ^2^Clinical Physics, Southern General Hospital, Greater Glasgow Health Board, Glasgow, UK

**Keywords:** ICP, TBI, autoregulation, compliance, non-invasive monitoring

## Abstract

Since Monro published his observations on the nature of the contents of the intracranial space in 1783, there has been investigation of the unique relationship between the contents of the skull and the intracranial pressure (ICP). This is particularly true following traumatic brain injury (TBI), where it is clear that elevated ICP due to the underlying pathological processes is associated with a poorer clinical outcome. Consequently, there is considerable interest in monitoring and manipulating ICP in patients with TBI. The two techniques most commonly used in clinical practice to monitor ICP are via an intraventricular or intraparenchymal catheter with a microtransducer system. Both of these techniques are invasive and are thus associated with complications such as hemorrhage and infection. For this reason, significant research effort has been directed toward development of a non-invasive method to measure ICP. The principle aims of ICP monitoring in TBI are to allow early detection of secondary hemorrhage and to guide therapies that limit intracranial hypertension (ICH) and optimize cerebral perfusion. However, information from the ICP value and the ICP waveform can also be used to assess the intracranial volume–pressure relationship, estimate cerebrovascular pressure reactivity, and attempt to forecast future episodes of ICH.

## Introduction

**Figure 1:**
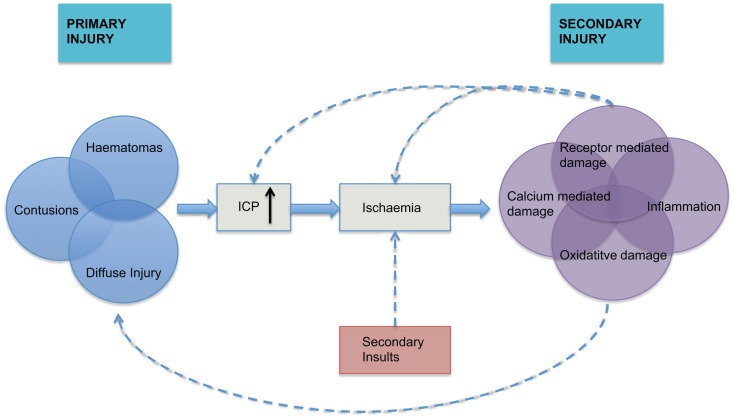
**The inter-relationship between primary and secondary injury in TBI is shown**. Secondary physiological insults can potentiate ischemia and lead to exacerbation of secondary injury. ICP = intracranial pressure, adapted from Mass et al. (2).

The pathophysiology of traumatic brain injury (TBI) can be divided into primary and secondary injury. The primary injury may include focal hematomas, contusions, or diffuse injury that leads to a cycle of hypoxic ischemic injury associated with inflammatory and neurotoxic processes (Figure 1). This secondary injury is exacerbated by secondary physiological insults such as hypoxia, hypo- or hypercarbia, hypotension, hyperthermia, and hypo- or hyperglycemia. A rise in intracranial pressure (ICP), or intracranial hypertension (ICH), is a secondary insult that can result from the primary injury, vascular engorgement, obstruction to cerebrospinal fluid (CSF) flow or cerebral edema. It is known to be associated with poorer outcomes (1), which has led to considerable interest in its monitoring and manipulation in patients who have suffered TBI.

Normal ICP in healthy adults is usually regarded as 5–15 mmHg (3) and in TBI an ICP of >20 mmHg is widely accepted as ICH (4). The principle aims of ICP monitoring in TBI are to allow early detection of secondary hemorrhage and to guide therapies that limit ICH. In addition, measurement of ICP and mean arterial pressure (MAP) allows calculation of cerebral perfusion pressure (CPP):

(1) CPP=MAP−ICP

Attempts can then be made to optimize CPP with the aim of preventing cerebral ischemia.

There is ongoing debate over the central role of ICP monitoring in the clinical management of TBI. This is particularly relevant in the context of a recent randomized controlled trial (RCT) that did not show an outcome benefit in patients undergoing ICP monitoring with a treatment threshold of 20 mmHg when compared to patients that were not monitored (5). The purpose of this review is therefore to reconsider some of the basic science underlying ICP monitoring and the ICP–volume relationship in adults. With this pretext, we will then support the arguments of other authors for the use of ICP as “more than a number” or a generic treatment threshold (6). Instead, the information within ICP trends and the ICP waveform can be used to provide individualized treatment thresholds and forecast future episodes of ICH.

## Concepts and Historical Perspective

### Intracranial contents

The Monro–Kellie hypothesis describes the relationship between the contents of the skull (7). In 1783, Monro published his observations that: the brain was enclosed in a non-expandable case of bone; the substance of the brain was nearly incompressible; the volume of the blood in the cranial cavity was therefore constant or nearly constant; and a continuous outflow of venous blood from the cranial cavity was required to make room for the continuous incoming arterial blood. Experiments performed by Kellie and Abercrombie supported these observations but they, like Monro, did not account for the role of CSF.

As the important role of CSF was recognized, the Monro–Kellie hypothesis was revised to its current form where with an intact skull, the sum of the volumes of the brain, intracranial blood, and CSF are constant. Therefore, an increase in one necessitates a decrease in one or both of the remaining two. As the brain parenchyma is essentially non-compressible, compensation is achieved through extrusion of CSF or venous blood.

### Intracranial pressure measurement

Lundberg systematically described the technique of continuous ICP monitoring using an intraventricular catheter in a series of 130 patients with suspected intracranial space occupying lesions (8). He then went on to confirm the feasibility of the technique in a series of 30 patients with TBI (9).

In his seminal paper, Lundberg identified three typical patterns of ICP fluctuation, which have come to be known as “A,” “B,” and “C” waves. A waves are steep rises in ICP to a plateau of 50 mmHg or more and are sustained for 5–20 min before falling rapidly. They represent a critical reduction in intracranial compliance. B waves occur with a frequency of 0.5–2 Hz and are rhythmic oscillations to 20–30 mmHg above the baseline but without a sustained period of ICH. C waves are not thought to be of pathophysiological importance, probably a reflection of Traube–Hering waves originating in the arterial pressure and are of much smaller amplitude to B waves.

While Lundberg and colleagues were developing the role of ICP monitoring in man, Langfitt’s group were examining primates to carefully characterize the transmission of pressure across the intracranial compartments (10, 11). The phenomenon of pressure underestimation was fully defined in experimental studies of extradural brain compression where progressive loss of transmission of ICP across the tentorial hiatus occurred, with the pressure in the posterior fossa and lumbar subarachnoid space progressively under-reading the ventricular pressure and eventually returning to normal pressure.

### Exploring the intracranial volume–pressure relationship

**Figure 2:**
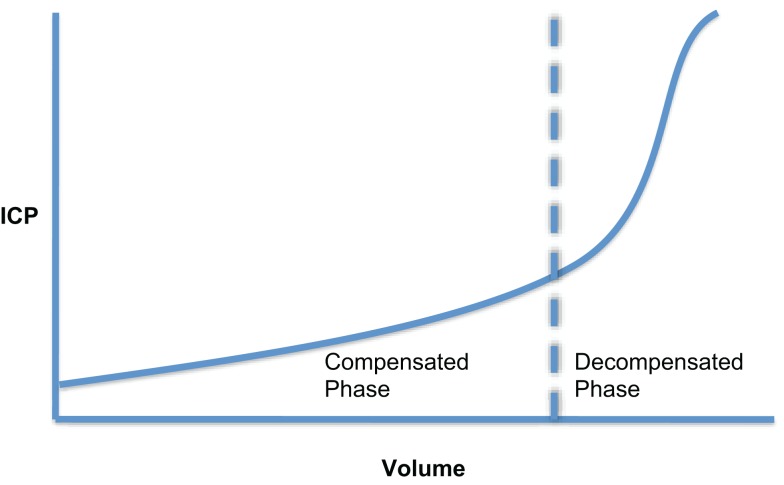
**Cerebral volume–pressure curve showing the exponential relationship between ICP and an increase in volume of one of the intracranial components**.

The intracranial volume–pressure curve demonstrates how small increase in volume of one of the intracranial components can be compensated by a reduction in CSF or blood volume (Figure 2). However, these compensatory measures are quickly exhausted and any subsequent increase in volume leads to an exponential increase in ICP. Measurement of this volume–pressure relationship is most often incorrectly referred to as intracranial compliance. According to conventional terminology, it should be referred to as elastance (change in pressure per unit change in volume, Δ*P*/Δ*V*) (12, 13). Due to the exponential nature of the volume–pressure relationship as depicted in Figure 2, being able to quantify elastance is attractive clinically as, in theory, it will increase during the volume compensation phase more rapidly than ICP and should therefore be predictive of impending volume decompensation.

The first full mathematical description of the craniospinal volume–pressure relationship was published by Marmarou in 1973 (14). Since then, several research groups have contributed physiological simulation models of ICP dynamics of varying complexity. These models aim to improve understanding of ICP pathophysiology and thus assist in the development of appropriate treatment strategies. A detailed comparative review on this subject has been provided by Wakeland and Goldstein (15). The early work of Marmarou and colleagues shall be discussed below as it provides an introduction to many important concepts surrounding ICP dynamics.

**Figure 3:**
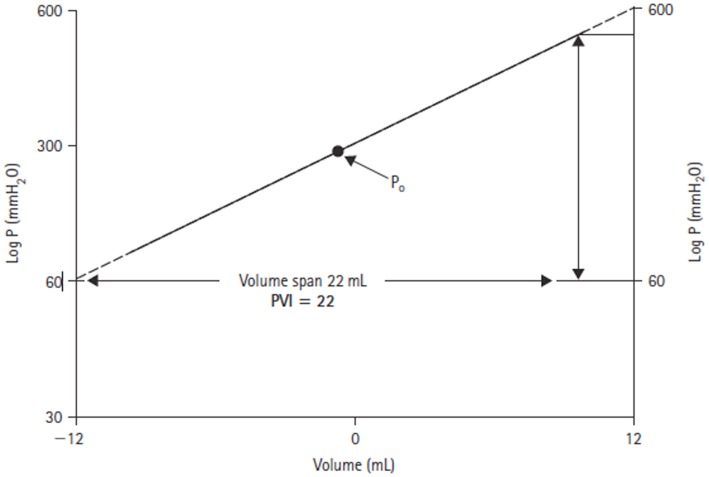
**Log ICP vs. intracranial volume relationship defined by Marmarou (14)**. The pressure–volume index (PVI) is the notional volume (milliliters), which when added to the craniospinal volume causes a 10-fold rise in ICP.

Through his interest in the pathological state of hydrocephalus, Marmarou developed a mathematical model of the CSF system that produced a general solution for the CSF pressure (14). The model parameters were verified experimentally in a series of experiments on adult cats (16). In these studies, the CSF pressure was measured both intracranially at the cisterna magna and in the lumbar subarachnoid space in response to bolus injections. Of particular note in this work, was the introduction of the pressure–volume index (PVI). Marmarou confirmed the non-linear relationship between changes in craniospinal volume and pressure. However, by plotting changes in volume against the log of pressure, a straight-line relationship could be defined (Figure 3). The slope of this line is termed the PVI and is the notional volume required to raise ICP 10-fold. Unlike elastance or compliance, the PVI characterizes the craniospinal volume–pressure relationship over the whole physiological range of ICP.

Calculation of the PVI by measuring the pressure change in response to a rapid injection or withdrawal of fluid from the subarachnoid space has previously been used both experimentally and clinically as a measure of craniospinal elastance (17–21). Shapiro found that a PVI reduced by 80% of control values was predictive of raised ICP in pediatric TBI (22). Similarly, Tans and Poortvliet measured PVI in adults with a range of brain injuries, including TBI, and demonstrated that a reduced index was associated with impending ICH (23).

**Figure 4:**
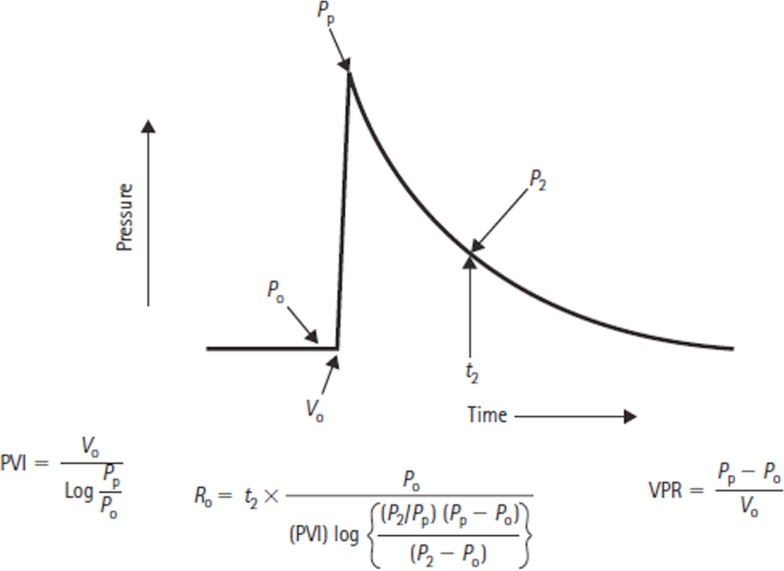
**Formulas for deriving the pressure–volume index (PVI), volume–pressure response (VPR), and the CSF outflow resistance (*R*_o_), where *P*_0_ is the baseline CSF pressure, *P*_p_ is the peak pressure resulting from a bolus volume injection *V*_0_, and *P*_2_ refers to the pressure point on the return trajectory at time*t*_2_**.

Marmarou’s mathematical model developed an improved understanding not only of craniospinal elastance but also of the inter-relationships of the static and dynamic processes of formation, storage, and absorption of CSF. Previously, Davson had demonstrated that by withdrawing CSF at the estimated rate of CSF production (approximately 0.3 ml/min), it was possible to determine the cerebral venous pressure (24). This value could then be substituted into the steady-state ICP equation:

(2) $$\text{ICP}=P_{\text{ssp}}+\left(I_{\text{f}}\times R_{\text{o}}\right)$$

where *P*_ssp_ is cerebral venous pressure, *I*_f_ is CSF formation rate, and *R*_o_ is CSF outflow resistance. Marmarou extended Davson’s work and his general solution for ICP allowed the derivation of an equation for CSF outflow resistance based on a bolus injection technique (Figure 4) (14, 16).

In TBI management, it is useful to know CSF outflow resistance when determining the etiology of raised ICP. In general terms, causes of ICH can be categorized into “vascular” and “non-vascular” mechanisms. Vascular mechanisms include active cerebral vasodilation due to stimuli such as increased arterial carbon dioxide levels or decreased CPP with intact pressure autoregulation, passive distension of cerebral vessels in the absence of autoregulation or venous outflow obstruction. Non-vascular mechanisms include increased brain mass due to cerebral edema or an expanding extradural, subdural, or intracerebral mass. A further non-vascular mechanism is an increase in CSF outflow resistance secondary to obstruction of the normal CSF pathway.

The importance of vascular factors and the state of cerebral blood flow (CBF) autoregulation as a determinant of craniospinal elastance was shown clearly by the work of Gray and Rosner (25, 26). The autoregulation of CBF will be discussed later, however, through a series of studies in adult cats, Gray and Rosner demonstrated that with CPP levels >50 mmHg, there was a linear increase in PVI with increasing CPP. Similarly, with CPPs below 50 mmHg, further reduction in CPP was also associated with increased PVI, as well as reduced CBF. This work illustrated that the PVI is a complex function of CPP and that the direction of the CPP–PVI relationship is dependent on whether CPP is above or below the autoregulatory range for CBF. The importance of the state of autoregulation on PVI has been supported recently by Lavinio et al. (27). In a series of brain-injured patients admitted to the intensive care unit (ICU), PVI results were significantly different if a transcranial Doppler (TCD) derived assessment of middle cerebral artery (MCA) flow velocity (FV) revealed defective cerebral autoregulation.

Despite the potential for providing valuable information on the ICP–volume relationship, the PVI is not routinely measured in clinical management of severe TBI. Variability between measurements is high because of the difficulty in rapid manual injection at a constant rate. As a result, an average of repeated measures is usually required. In addition, there is an infection risk associated with injecting fluid into the subarachnoid space via an intraventricular catheter (28–30) and a risk of provoking secondary ICP rises following injection as a consequence of vasodilation (31).

Thus, an interest in deriving estimates of the ICP–volume relationship indirectly through analysis of the ICP waveform has become a research focus.

### ICP waveform

**Figure 5:**
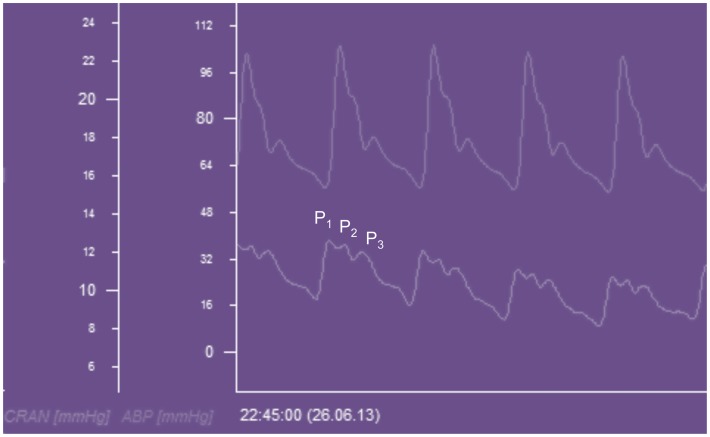
**ICP waveform recorded from a Raumedic intraparenchymal catheter and displayed beneath an arterial waveform recorded from the radial artery in a patient with TBI**. CRAN = intracranial pressure, ABP = arterial blood pressure, *P*_1_ = percussion wave, *P*_2_ = tidal wave, *P*_3_ = dicrotic wave.

The ICP waveform has three consistent peaks that are related to the arterial pulse waveform (Figure 5), although their exact etiology is the subject of some debate (32). Avezaat and van Eijndhoven systematically studied the ICP waveform pulse amplitude (ICP_plse_) as a measure of craniospinal elastance (31, 33). In recognition of the limitations of the PVI, related to the need for volume injection or withdrawal, they exploited the fact that with each cardiac cycle there is a pulsatile increase in cerebral blood volume. This is the equivalent of a small intracranial volume injection, and the ICP_plse_ is the pressure change in response to that volume increment and should consequently be directly related to the craniospinal elastance (d*P*/d*V*). Therefore, as craniospinal elastance increases (compliance decreases) the ICP_plse_ should increase. The observation that as ICP increases so does the amplitude of the ICP pulsations is not a new one, having been first described in 1866 by Leyden (34).

**Figure 6:**
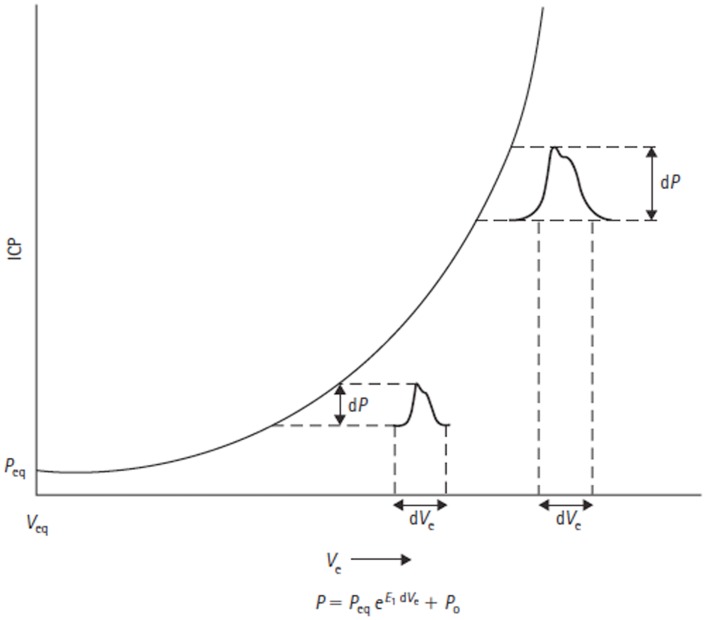
**Volume–pressure relationship and equation are shown**. Adapted from Avezaat and Van Eijndhoven (31). Craniospinal volume–pressure relationship demonstrating that for the same increase in craniospinal volume (d*V*_e_) the ICP pulse amplitude (d*P*) increases when total craniospinal volume (*V*_e_) increases. This is due to the exponential nature of the curve, which is described mathematically by the equation below the figure, where *E*_1_ is the elastance coefficient and determines the elastance at a given pressure. *P*_eq_ = intracranial equilibrium pressure, *P*_0_ = ICP at zero elastance.

The mathematical description of the exponential craniospinal volume–pressure relationship was extended by Avezaat and Van Eijndhoven through the introduction of a constant term *P*_0_ into the pressure–volume equation. Primarily for mathematical convenience, this term shifts the volume–pressure curve as a whole up or down its axis, which allows for correction of pressure transducer reference position and postural changes. Mathematically, *P*_0_ is the pressure at zero elastance (Figure 6) and must therefore have physiological significance as a determinant of the normal intracranial equilibrium pressure (*P*_eq_). Löfgren showed that alterations in central venous pressure (CVP) can shift the pressure–volume curve up or down its axis (35), which would suggest CVP may be a factor determining *P*_0_.

To allow validation of ICP_plse_ as a measure of elastance, Avezaat and Van Eijndhoven compared the relationship of ICP_plse_ versus ICP and elastance, as invasively measured by volume injection, versus ICP. This was performed in a series of 58 patients undergoing ICP monitoring for a variety of neurosurgical indications. A linear relationship between both ICP_plse_ and ICP and invasively measured elastance and ICP was confirmed, supporting the mono-exponential relationship between intracranial volume and ICP. However, the correlation between these relationships was weak.

**Figure 7:**
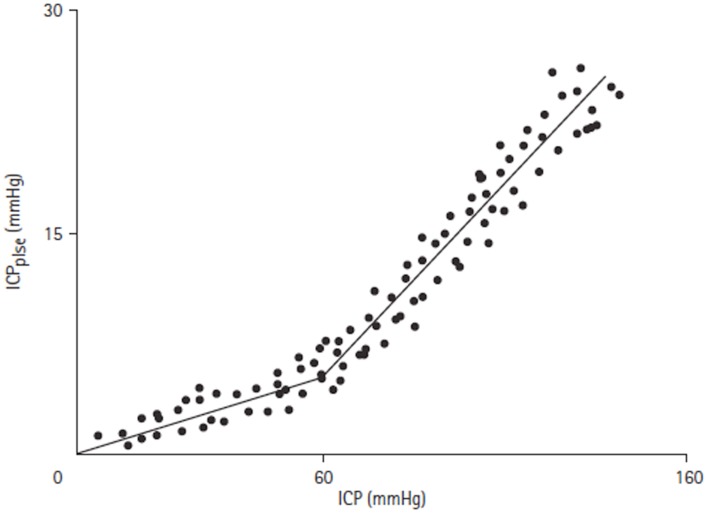
**ICP_plse_ versus ICP relationship [adapted from Avezaat and van Eijndhoven (31)]**. ICP_plse_ plotted against ICP, demonstrating a direct linear relationship. A breakpoint occurs at an ICP of approximately 60 mmHg where the slope of the relationship increases.

Of particular note in the above study was the observation that there was a disproportionate increase in ICP_plse_ during plateau waves, which was felt secondary to an increase in d*V* due to defective cerebral vascular muscle tone. To explore this phenomenon further they monitored ICP_plse_ while manipulating ICP in adult dogs by inflating an epidural balloon. They found that the ICP_plse_ increased linearly with ICP up until a pressure of 60 mmHg (Figure 7). At this pressure a breakpoint occurred and the ICP_plse_ increased more rapidly with increasing ICP. It was postulated that the breakpoint marked the loss of CBF autoregulation, which will be dealt with in more detail below.

The major limitation of using ICP_plse_ as a measure of craniospinal elastance (dP/dV) is the need to assume that the volume of pulsatile blood (dV) is constant. This is unlikely to be the case in severe brain injury because of the associated cardiovascular complications. Therefore, the clinical utility of this technique is limited unless the pulsatile blood volume can be controlled for.

### Cerebral autoregulation

#### Principles of cerebral autoregulation

**Figure 8:**
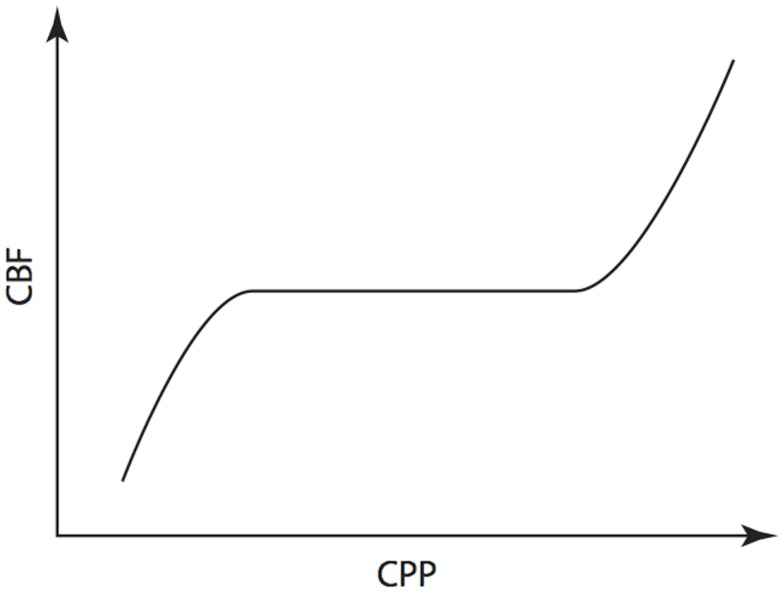
**Cerebral autoregulation**. Illustration of the maintenance of cerebral blood flow across a range of cerebral perfusion pressures.

As suggested earlier, one of the principle clinical reasons to monitor ICP is to allow calculation of CPP. This is useful because, in theory, maintenance of a CPP within the limits of cerebral autoregulation will result in maintenance of adequate CBF to meet the metabolic demands of the brain (36). Regulation of flow is achieved by active dilation and constriction of cerebral arterioles in response to changes of CPP and is illustrated in Figure 8. A number of physiological mechanisms are known to be involved in this process and Hamner and Tan have recently quantified the relative contributions of sympathetic, cholinergic and myogenic mechanisms (37). By measuring CBF while manipulating CPP, and utilizing pharmacological blockade of the three mechanisms, they were able to demonstrate the effect that each had on cerebral autoregulation in healthy volunteers. Of note, they found that 38% of the pressure-flow relationship was unexplained by these mechanisms, implying that others must also be important.

The physiological range of autoregulation, is regarded as 50 to 150 mmHg in healthy adults (36). When CPP is below the lower limit of the autoregulatory range, vessels within the arterial-arteriolar bed tend to passively vasoconstrict. Conversely, when CPP is above the upper limit, passive vasodilation occurs. Using measures of CBF including intra-arterial xenon clearance (38) and TCD FV of the MCA (39), it has been demonstrated that disordered cerebral autoregulation occurs after severe TBI and is associated with worse outcome.

#### Mathematical models of autoregulation

As discussed above, there is an extensive literature on the mathematical modeling of ICP dynamics. Several of these models incorporate descriptions of cerebral autoregulation. The models can be primarily physiology based, and aim to improve our understanding of the interaction between ICP dynamics and autoregulation, or they can have a more statistical basis and aim to provide an index of the state of autoregulation. Examples of each type of model shall be considered in turn below.

#### Physiological models of autoregulation

**Figure 9:**
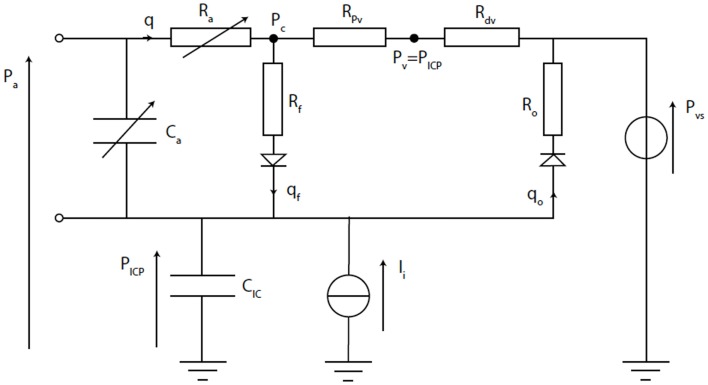
**Electrical equivalence circuit of the Ursino model (42)**. CBF (*q*) enters the intracranial space at systemic arterial pressure (*P*_a_). It is subject to arterial resistance (*R*_a_) and the cerebrovascular bed has some storage capacity (*C*_a_). CBF is then through proximal (*R*_pv_) and distal (*R*_dv_) venous resistance. Venous pressure (*P*_v_) is assumed to equal ICP (*P*_ICP_). *P*_ICP_ is dependent upon the volume stored in intracranial compliance (*C*_IC_). This is dependent upon blood volume in *C*_a_, CSF inflow (*q*_f_) through inflow resistance (*R*_f_), and CSF outflow (*q*_o_) through outflow resistance (*R*_o_), which is itself dependent upon venous sinus pressure (*P*_vs_). The system can be disturbed by mock CSF injection (*I*_i_).

Ursino and Lodi published a simplified mathematical model of the interaction between ICP and cerebral hemodynamics that is a cut down version of Ursino’s earlier work (40–42). The model is a two compartment model, which incorporates the hemodynamics of the arterial–arteriolar cerebrovascular bed, CSF production, and reabsorption processes, the pressure–volume relationship of the craniospinal compartment, and a Starling resistor mechanism for the cerebral veins (Figure 9). Importantly, it includes a parameter to account for the maximum autoregulatory gain. Using this model in a series of 20 patients with severe TBI, Ursino et al. were able to classify the state of cerebral autoregulation and predict the response of ICP to PVI testing (41).

**Figure 10:**
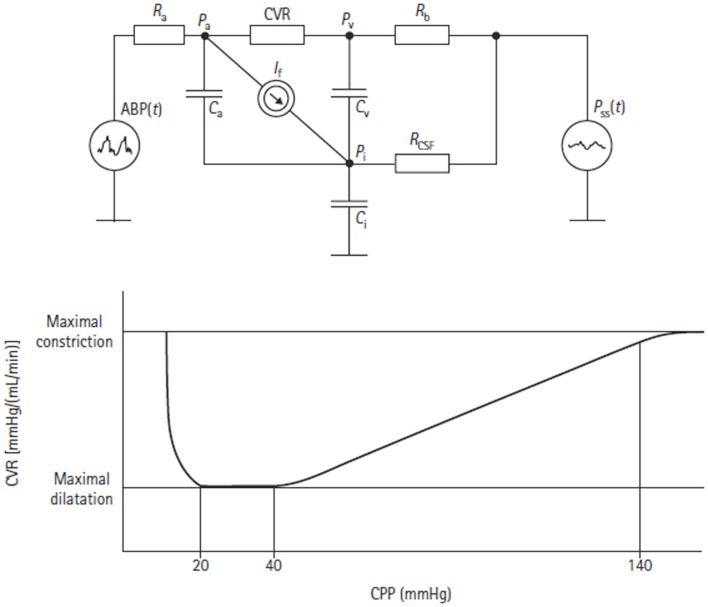
**Electrical equivalent circuit of the Czosnyka model (43)**. This figure Illustrates the presence of three storage compartments (*C*_a_ = compliance of the great cerebral arteries, *C*_v_ = compliance of capillaries, and small veins, *C*_i_ = compliance of the CSF containers). Other parameters are arterial blood pressure (ABP), cerebral arterial pressure in the small arteries (*P*_a_), pressure in the cortical veins (*P*_v_), ICP (*P*_i_), sagital sinus pressure (*P*_ss_), resistance of great cerebral arteries (*R*_a_), cerebrovascular resistance (CVR), resistance of cortical and bridging veins (*R*_b_), CSF outflow resistance (*R*_CSF_), and CSF secretion (*I*_t_). The lower figure shows the autoregulatory relationship between CVR and CPP as predicted by the model.

Czosnyka has also proposed a compartment model of CBF and CSF circulation (43). It is a three compartment model that consists of two vascular storage compartments (arterial and venous) and one CSF storage compartment (Figure 10). Again, this model is able to simulate the state of autoregulation. Using data taken from 82 patients admitted to ICU with moderate and severe TBI, comparison was made between measured clinical responses and simulated model responses to events such as carotid artery compression, systemic arterial hypotension, and ICH. The mathematical modeling results were found to be helpful with interpretation of the clinical phenomena. In particular, the model demonstrated that the correlation between arterial blood pressure (ABP) and ICP is dependent on the state of autoregulation. Czosnyka exploited this fact in development of the pressure reactivity index (PRx), which will be discussed in the following section.

**Figure 11:**
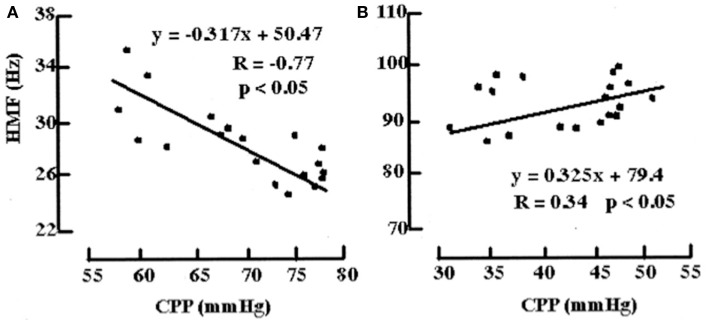
**Examples of the relationships between HMF and CPP during challenge with norepinephrine before and after fluid percussion injury (FPI)**. **(A)** Before FPI. Challenge with norepinephrine resulted in a response consistent with active vasoconstriction in that a negative correlation value (*R* = -0.77) and negative slope (*m*) of the regression line (*m* = -0.317 Hz/mmHg) between HMF and CPP were demonstrated. **(B)** After FPI. Consistent with passive vasodilation, challenge with norepinephrine resulted in positive correlation values (*R* = 0.34) and slope of regression line (*m* = 0.325).

An example of a model bridging the gap between physiological and more statistical or data-driven models of autoregulation is provided by Daley et al. (44). Using a definition of cerebrovascular pressure transmission provided in the above model by Czosnyka, the technique of modal analysis was applied. That is, a calculation of the highest modal frequency (HMF) at which energy is transferred from ABP to ICP. The HMF is calculated using an autoregressive moving average (ARMAX) technique and has been tested in a piglet model of raised ICP. It was found that when cerebral autoregulation was intact, a rise in CPP led to a decrease in HMF. In contrast, when there was autoregulatory impairment, a rise in CPP was met with an increase in HMF (Figure 11). Similar results have been seen in patients admitted to ICU with severe TBI (45).

#### Data-driven indices of cerebral autoregulation

The most systematically investigated statistical approaches to autoregulatory assessment, using ICP as an input parameter, is the PRx described by Czosnyka et al. (46). It is based on the hypothesis that naturally occurring slow oscillations of ABP can be used to evaluate the cerebrovascular reactivity. In theory, when pressure reactivity is intact, an increase in ABP would result in cerebral vasoconstriction and a reduction in ICP (negative PRx). Conversely, when pressure reactivity is absent, an increase in ABP would result in a passive rise in ICP (positive PRx). Pressure reactivity has a complex relationship with cerebral autoregulation rather than the expressions being analogous.

The PRx is a moving correlation coefficient between 40 consecutive samples of values for ABP and ICP averaged over a period of 5 s. By employing this averaging interval, most of the frequency changes above 0.2 Hz in the ABP and ICP recordings are filtered out. In addition, Nyquist’s sampling theorem dictates that the highest frequency that can be represented by a signal sampled every 5 s is 0.1 Hz or 6 oscillations/min. As a result, the dynamical system relationship between ABP and ICP cannot be precisely defined by PRx.

Nevertheless, PRx has been found to be a very useful tool in clinical research. In TBI, it has been demonstrated to provide a reliable index of cerebral autoregulation as validated by TCD (46) and PET (47) derived measurements. Clinical observations show that the PRx is high both during the occurrence of plateau waves and also during refractory raised ICP (48). In addition, the PRx has been used to guide proposed therapies and calculation of an “optimal CPP” for the management of patients with TBI (49).

#### Comparison of mathematical models of cerebral autoregulation

Despite illustrating a number of the approaches that can be taken, this is by no means an exhaustive list of models of CBF autoregulation. It is not clear which approach is most clinically practical or useful. The models take different input parameters and yield different output indices, thus making comparison difficult. In an attempt to address this issue, Shaw et al. re-worked and normalized three of the models so that a fair evaluation could be made on a standardized dataset of ABP, ICP, and MCA FV readings taken from piglets pre- and post-fluid percussion injury (50, 51). The state of autoregulation predicted by the models could then be compared to changes in pial artery diameter as a direct measure of autoregulation. One of the interesting conclusions from this work was that before application of a number of optimization approaches, none of the models performed particularly well. Overall, Ursino’s physiological model performed best and after optimization of the data-driven models, Daley’s HMF autoregulatory index performed marginally better than Czosnyka’s PrX. This work is limited by the use of only one small dataset for comparison. What is certain, however, is that further studies comparing autoregulatory methods and optimization approaches are warranted before widespread clinical adoption of a standardized autoregulation model is possible.

In recognition of this challenge, an international group of those working in both experimental and clinical autoregulation research have setup a new consortium called the “Cerebral Autoregulation Network” or CAR-Net (52).

## Current Controversies

### Should ICP be monitored in severe TBI?

Monitoring of ICP has become a standard of care in severe TBI and its use is supported by internationally applied guidelines. The Brain Trauma Foundation recommends that ICP should be monitored in all salvageable patients with severe TBI and an abnormal computed tomography (CT) scan (53). Further, they recommend that monitoring should then be used to target ICP <20 mmHg and CPP 50–70 mmHg.

The evidence for and against ICP monitoring in TBI has been appraised in several excellent reviews (54–56). Supporting the use of ICP monitoring are retrospective comparisons of historical cohorts at the same center suggesting that protocols incorporating ICP monitoring improve outcome (57, 58). Similarly, there has been an association between centers monitoring ICP more frequently and better outcome (59). In contrast, a retrospective comparison of two trauma centers revealed an increase in therapy levels without an improvement in outcome in the center that monitored ICP (60).

On the basis of the wealth of conflicting evidence, there was demand for an RCT to assess the impact of ICP monitoring on clinical outcomes. An RCT of 324 patients with severe TBI was subsequently performed in Latin America (5). Patients were assigned to protocolized therapy directed by either ICP monitoring or clinical examination and imaging. There was no difference between groups in the primary outcome of a composite of survival time, impaired consciousness, and functional status at 3 and 6 months and neuro-psychological status at 6 months.

This study has been subject to extensive discussion and editorial review (61–64) by the lead investigator (65). Irrespective of the applicability of the findings to the routine practice of ICP monitoring in severe TBI, the results certainly strengthen the argument for more clearly defining the use of ICP targeting strategies as part of an individualized and multimodal approach to this patient group.

### What modality should be used to monitor ICP?

#### Introduction

The two techniques most commonly used in clinical practice to monitor ICP are via an intraventricular or intraparenchymal catheter with a microtransducer system. Both of these techniques are invasive and are thus associated with complications such as hemorrhage and infection. For this reason, significant research effort has been directed toward development of a non-invasive method to measure ICP.

#### Intraventricular catheter

**Figure 12:**
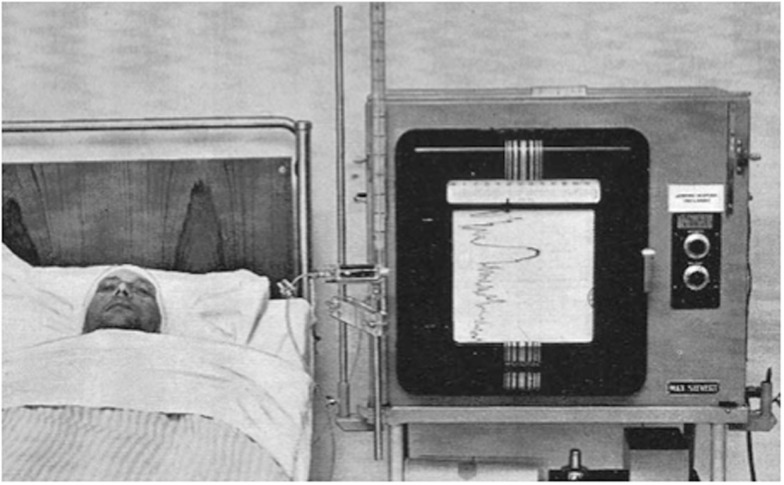
**Image from Lundberg’s 1960 publication on Continuous recording and control of ventricular fluid pressure in neurosurgical practice (8)**.

Following Lundberg’s description of the use of intraventricular catheters for the continuous measurement of CSF pressure (8), the technique has remained the gold standard for ICP monitoring (66). It is performed by inserting a catheter into either lateral ventricle through a frontal burr hole. In 1960, Lundberg was already using electronic measurement equipment by connecting the ventricular cannula via a strain gage transducer to a potentiometer recorder (Figure 12). In modern practice, the ventricular catheter can similarly be connected to an external strain gage or the ICP waveform can be transduced via fibreoptic or micro-strain-gauges within the catheter itself.

An advantage of measuring ICP using an intraventricular catheter is the opportunity to perform drainage of CSF as an ICP lowering therapy. It is also possible to recalibrate the monitor while *in situ* and thus retain accuracy for several days of monitoring. However, as suggested above, the technique is not without risk. It can be technically difficult in the case of ventricular effacement or midline shift. There is a risk of CSF infection but this can be kept to as low as 10% with a “Bundle” based approach to care (67). The incidence of hemorrhage following ventriculostomy is around 1%, although the number requiring surgical evacuation is likely to be lower (66).

#### Intraparenchymal catheter

In cases where intraventricular ICP monitoring is not possible, or in many centers as the preferred technique, an intraparenchymal device can be placed. The principle difference with the intraparenchymal devices is the inability to recalibrate them following insertion with the consequent problem of zero drift. Bench testing of devices using both fibreoptic tips (Camino OLM ICP monitor; Camino Laboratories, San Diego, CA, USA) and micro-strain-gauges (Codman Microsensor ICP Transducer; Codman & Shurtlef Inc., Randolph, MA, USA) have shown 24 h zero drift of <0.8 mmHg (68). Similarly, laboratory testing of an intraparenchymal device incorporating a micro-strain-gauges with a complete Wheatstone bridge circuit incorporated into the tip (Raumedic AG, Münchberg, Germany), demonstrated a mean zero drift of 0.6 mmHg at 5 days (69). However, in the more demanding clinical environment, a multicentre evaluation concluded that the zero drift rate remained a concern and catheter performance was similar to that of other manufacturers (70).

Intraparenchymal ICP monitoring devices are typically placed via a small burr hole into the white mater of the non-dominant frontal hemisphere. These devices measure a compartmentalized local pressure and significant supratentorial pressure gradients have been demonstrated between monitoring ipsi- and contralateral to the side of focal hematomas (71).

#### Non-invasive ICP monitoring

For a non-invasive measure of ICP to replace the commonly used invasive measures above, it must provide an accurate absolute measure of ICP that can be performed continuously at the bedside. There is no current technique that satisfies these criteria. An in depth review of all of the available technologies is out with the scope of this article and has been covered in detail elsewhere (72–74). Techniques considered include imaging based studies using CT and magnetic resonance imaging (MRI), TCD sonography, near-infrared spectroscopy (NIRS), tympanic membrane displacement (TMD), visual-evoked potentials (VEPs), measurements of optic nerve sheath diameter (ONSD), and other measurements of the optic nerve, retina, and pupil. Of these, approaches using TCD and ONSD have perhaps received the most clinical interest.

Using low frequency TCD, it is possible to measure FV in the MCA (75). Several authors have published equations using the MCA FV metrics of peak systolic velocity (PSV), mean FV (mFV), end diastolic velocity (EDV), and pulsatility index (PI, PSV–EDV/mFV) to estimate ICP and CPP.

Schmidtt et al. examined 25 patients admitted with severe TBI and calculated non-invasive CPP (nCPP) as MAP × EDV/mFV + 14 mmHg (76). For these patients, 81% of 1 min averages of nCPP (*n* = 12 275) were different from invasively measured CPP (iCPP) by <10 mmHg. In 81 brain-injured patients, including 21 with TBI, Bellner et al. calculated non-invasive ICP (nICP) as 10.93 × PI − 1.28 (77). Bland and Altman analysis of all measurements (*n* = 658) revealed that the difference between nICP and invasively measured ICP was <4.2 mmHg for 95% of measurements. Edouard et al. calculated nCPP as [mFV/(mFV − EDV)] × (MAP − DAP) in patients with severe TBI and bilateral injury (78). In 10 patients, repeated measurements were made during their clinical course (*n* = 89) and a significant correlation was found between nCPP and iCPP. However, in a further 10 patients in whom hypercapnia was induced, the strength of this correlation was reduced.

The performance of the above three equations in estimating ICP was compared in 45 patients with severe TBI by Brandi et al. (79). Under standardized conditions, including continuous sedation, normocapnia and normothermia, daily nICP measurements were compared to ICP measured using an intraparenchymal device. On the basis of Bland and Altman analysis, the authors concluded that the equation by Bellner et al. (77) was superior in assessing nICP. However, as has been noted elsewhere (54), the Bellner equation failed to predict all cases of ICH in this series and is therefore not likely to be clinically useful as a screening test in TBI.

Like TCD measurements, assessment of ONSD using ultrasound potentially provides a simple bedside screening test for ICH in TBI. The technique exploits the fact that the optic nerve is part of the central nervous system and therefore, a rise in ICP will be transmitted through the CSF surrounding the nerve. Several studies comparing ultrasound derived ONSD assessment to iICP (80–85) have been included in a recent meta-analysis (86). This was limited by the fact that it included only 231 patients, 89 of whom had suffered TBI. However, using the ONSD thresholds reported in the individual studies, the pooled sensitivity and specificity to detect ICH were 90 and 85%, respectively. Dubourg et al. are now collecting data for an individual patient data meta-analysis with the objective of defining the cut-off value for ultrasound-derived ONSD in the detection of ICH (87).

### Should ICP or CPP be the target?

Whatever modality is chosen to monitor ICP in severe TBI, the clinician must then decide whether to primarily target therapy at attempting to optimize CPP or lower ICP. CPP oriented therapy, as proposed by Rosner et al. (88), requires pressure autoregulation and the ability to manipulate CPP within the autoregulatory range. During intact pressure regulation, increases of CPP cause constriction of the arterial–arteriolar vascular bed and lowering of ICP by a reduction in cerebral blood volume. In addition, the resulting reduction of pre- and post-capillary pressure decreases fluid filtration and increases absorption, thus reducing brain edema. However, the application of CPP oriented therapy when autoregulation has been lost may result in an imbalance of Starling forces at the capillaries leading to increased net fluid filtration and further brain injury by increased production of vasogenic edema.

Avoiding vasogenic edema is one of the underlying tenets of the “Lund” approach to management of severe TBI based on lowering ICP (89, 90). Asgeirsson et al., working at the University Hospital of Lund, described a protocol aimed at inducing transcapillary fluid absorption through reduction of hydrostatic capillary pressure and preservation of normal colloid osmotic pressure. This included pharmacological interventions such as the reduction of systemic hypertension with metoprolol and clonidine, and precapillary vasoconstriction with dihydroergotamine.

In an attempt to determine whether an ICP or CPP based approach was preferable, Roberston et al. conducted an RCT in 189 patients admitted with severe TBI. Patients were randomized to an ICP based protocol or a CBF based protocol. The major differences between the protocols were the CPP targets (>50 mmHg in the ICP group and >70 in the CBF group) and the option to treat ICH with hyperventilation in the ICP group. In terms of the primary outcome of this study, cerebral ischemia as measured by jugular venous desaturations, the CBF based protocol was associated with a lower risk of ischemia. However, this did not translate into improved neurological outcome and indeed was associated with an increased frequency of systemic complications such as adult respiratory distress syndrome (ARDS).

It is likely that the choice of ICP or CPP based approach to ICU management of severe TBI should be made on an individual patient basis. For this to be possible, the state of autoregulation needs to be assessed.

**Figure 13:**
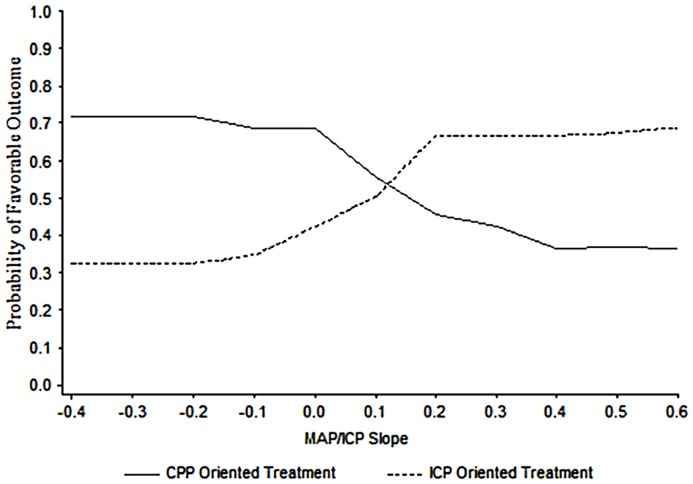
**BANN generated probability distribution plots for the mean likelihood of a favorable clinical outcome for patient populations managed in two different centers**. In this data, the optimal point at which to switch from one treatment strategy to the other in a given patient is at an MABP/ICP trend with a slope of approximately 0.13. Taken from Howells et al. (91).

Support for the clinical utility of a PRx type index has been provided by Howells et al. (91). The approach of two neurosurgical ICUs to ICP management in TBI was compared using a PRx-based index, averaged over many hours per day, and a machine learning Bayesian Neural Network (BANN) model, which predicted the probability of good or bad clinical outcome. In one center, the predominant management approach was CPP-targeted therapy and in the other, the approach was ICP-targeted therapy. The model showed that not only pressure reactivity was related to clinical outcome but also that its relationship to outcome was management approach dependent (Figure 13). From this data, a principally CPP-targeted approach was more successful when pressure reactivity was intact, while a principally ICP targeted approach was more successful when pressure reactivity was impaired. Of course, there could be other factors influencing clinical outcome that were not considered in the analysis. Nevertheless, it is compelling evidence for what appears to be common sense: a management strategy that considers the brains’ ability to regulate its blood flow is more successful than one that does not.

## Future Directions

### Introduction

The field of ICP research is a wide ranging one and, to date, has been the subject of 15 international symposia embracing such diverse disciplines as neurosurgery, intensive care, anesthesia, radiology, biophysics, electronic and mechanical engineering, mathematics, and computer science (92). This multidisciplinary and collaborative approach is highlighted by research groups such as International Mission for Prognosis and Analysis of Clinical Trials in TBI (IMPACT) (93), Brain Monitoring with Information Technology (BrainIT) (94), and the recently funded CENTER-TBI project (95).

At present, there is no level 1 evidence to support the targeting of a specific ICP or CPP using clinical interventions. This may change with ongoing RCT. For example, Eurotherm3235 is assessing titrated hypothermia to treat ICH (79, 80) and RESCUEicp is evaluating the role of decompressive craniectomy in treatment of uncontrollable ICH (96). In parallel to these trials, there is considerable effort to extract more information, rather than simply a generic threshold value, from the ICP signal and use this to provide patient-specific targets and to forecast secondary ICP insults. In addition, there is ongoing effort to develop novel non-invasive techniques to measure ICP and thus widen its clinical application. Some key areas of current research shall be discussed below.

### Individualized ICP and CPP targets

As an alternative to using a universal CPP threshold for all TBI patients, a more dynamic patient tailored CPP target, based upon the autoregulation capacity of the cerebral vasculature, has been proposed. In retrospective analysis, Steiner et al. (49) demonstrated that by plotting PRx against CPP for the entire monitoring period, a “U-shaped” curve could be produced in about 60% of patients. The CPP corresponding to the minimum PRx was taken to represent the optimal CPP (CPPopt) for each patient. Patients who were managed with CPPs closer to CPPopt were more likely to have a good outcome.

The feasibility of using PRx to prospectively calculate CPPopt in TBI patients in a clinical environment has subsequently been demonstrated by Aries et al. (97). Using a 4-h moving window, updated every minute, CPPopt could be calculated for 55% of the monitoring period. Again, patients were more likely to have a good outcome if their actual CPP deviated less from CPPopt.

In similar work, Lazaridis et al. (98) have used PRx to identify patient-specific ICP thresholds in TBI. By plotting PRx against ICP for the entire monitoring period, the threshold ICP was taken to be that at which the PRx was consistently >0.2. It was possible to calculate a threshold ICP in 68% of patients. Time spent above an individually calculated ICP threshold was more strongly predictive of mortality than using a generic threshold of 20 or 25 mmHg. This further supports the concept of patient-specific targets of ICP or CPP in the management of TBI.

**Figure 14:**
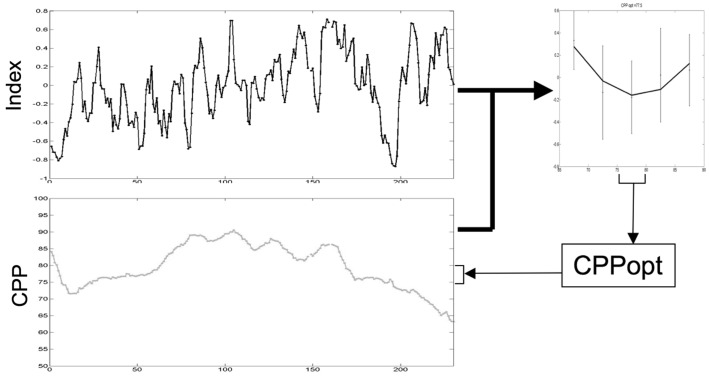
**Example of an optimal CPP range (CPPopt) derived from the most recent 4-h CPP and autoregulation index values**.

However, calculation of PRx and most other measures of autoregulation require high frequency data (>50 Hz) sampling. Capturing and processing this data frequency is not routine in many NICUs. Consequently, Depretiere et al. have developed a new index of cerebrovascular reactivity that requires only minute-by-minute data sampling (99). Known as LAx, the index is the moving median of minute-by-minute ICP/MAP correlation coefficients over different time intervals (3–120 min). They demonstrated that not only does it correlate with PRx and GOS but also is able to produce a CPPopt recommendation. DATACAR (Dynamic Adaptive Target of Cerebral Autoregulation) combines different LAx values and time windows in a weighted manner to issue a CPPopt recommendation (Figure 14). They observed significant differences between PRx-based and LAx-based CPPopts. DATACAR was able to issue a CPPopt recommendation in 92% of monitoring time, as opposed to 44% for PRx-based CPPopt.

Certainly, a method for continuous and robust determination of a patient’s optimal CPP that can work with normal NICU data capture rates, is an attractive concept. A prospective study comparing a number of these indices is warranted. These developments show clearly the benefits possible through the combination of sharing and analysis of large ICU datasets with the development and application of mathematical models.

### Prediction of secondary ICP insults

An interesting approach to forecasting ICH is based on preceding changes to waveform morphology. In recognition that most clinical decision making only takes into account the mean ICP, Hu and colleagues have proposed a technique for automatically extracting useful information from the ICP waveform (100). Morphological clustering and analysis of continuous ICP (MOCAIP) detects the P_1_, P_2_, and P_3_ peaks within the ICP waveform. The technique was developed and validated using an annotated database of ICP waveforms collected from 66 patients admitted to an adult hydrocephalus center. For every 3 min section of ICP recording, the MOCAIP algorithm performs beat-by-beat pulse detection followed by pulse clustering to generate a dominant ICP pulse. Artifactual pulses are removed prior to the detection and optimal designation of pulse peaks. This process has been generalized as MOCAIP++ and validated on a larger dataset collected from 128 patients (101).

The application of MOCAIP to ICP monitoring in TBI has been demonstrated (102). In a dataset from 66 patients, including 23 admitted with TBI, ICP pulse morphological metrics were correlated with low CBF as measured by an intravenous ^133^Xenon clearance technique. Of particular interest, was the association of an elevated P_3_ peak and low CBF. However, in this study, the correlation of pulse morphological metrics to low CBF was less in the TBI patients than in those admitted with other diagnoses such as subarachnoid hemorrhage.

In the first efforts to use MOCAIP analysis to forecast episodes of elevated ICP, an ICP waveform dataset recorded from 34 patients presenting with suspected idiopathic ICH, CSF shunts, and Chiari malformation was evaluated (103). Using 24 metrics of the ICP waveform, it was possible to classify recording segments as either control or pre-IH prior to episodes of elevation of ICP to >20 mmHg over a period of at least 20 min. This was done with a sensitivity of 37 and 21% and specificity of 99 and 99% for 5 and 20 min, respectively. These results are encouraging but may not generalize to TBI because of the difference in underlying pathophysiological mechanisms.

An alternative approach to prediction of ICH, which has been developed using data collected from patients admitted to NICU with TBI, is through the use of Gaussian processes (104). Using 4 h windows of minute-by-minute recordings of ICP and MAP, Guiza et al. generated over 1000 potential dynamic predictors from which a subset of 73 was selected. These included median values for non-overlapping time intervals, measures of variability, clustering of values based on their trajectory, frequency domain analysis, and correlation of ICP with MAP. Gaussian processes are a machine-learning algorithm that generates a probabilistic prediction based on the known outcomes of similar data instances. The model was developed in a cohort of 178 patients to predict 30 min in advance of an elevation of ICP to >30 mmHg over a period of at least 10 min. It was then evaluated in a further cohort of 61 patients achieving a sensitivity of 82% and specificity of 75%.

Future predictive models may incorporate both ICP waveform features and dynamic predictors to optimize their predictive capacity. The value of these predictions would then need to be assessed by providing them to clinicians and formally assessing the impact on patient management and outcome.

### Innovative non-invasive ICP monitoring

As suggested above, no methodology in current clinical use provides an accurate absolute measure of ICP. A novel technique, which provides an absolute value of ICP, has recently been described by Raguaskas et al. (105). A two-depth TCD device is used to identify the intracranial and extracranial components of the ophthalmic artery (IOA and EOA). Following the assumption that the Doppler waveform of the IOA is dependent on compression by ICP and that of the EOA by externally applied pressure (*P*_e_), a ring cuff is applied to the orbit and automatically inflated from 0 to 28 mmHg in 4 mmHg steps. The *P*_e_ at which the waveforms of the IOA and EOA are identical is taken to represent the ICP. A comparison study of this technique to CSF pressure measured by lumbar puncture was performed in 62 patients presenting to a neurology clinic, including 37 with suspected IIH and 20 with multiple sclerosis. For invasively measured CSF pressures in the range of 4–24, the non-invasive technique achieved a 98% confidence interval for the absolute error of ±4 mmHg.

In a study of a similar group of patients, the two-depth TCD technique was compared to the ONSD technique in its ability to predict raised CSF pressure as measured by LP (106). Using a CSF pressure threshold of 14.7 mmHg, and an ONSD cut-off of 5 mm, the two-depth TCD technique outperformed the ONSD technique with sensitivities of 68 and 37% and specificities of 84 and 59%. Clearly, neither of these techniques could be used for clinical decision making at these thresholds.

Further work is required to confirm the safety of the innovative two-depth TCD technique in terms of pressure effects on the globe and exposure of the lens to Doppler US. The applicability of the technique to the TBI population and across a wider range of ICP values has yet to be demonstrated.

## Conclusion

Despite the fact that ICP monitoring in TBI has become a standard of care, there is no level 1 evidence to support its use in targeting generic ICP thresholds. However, there can be little doubt that investigation of ICP and the ICP–volume relationship has led to an improved understanding of cerebral physiology. It is now time to exploit this knowledge and integrate ICP monitoring into a multimodality and individualized approach to care. Future RCTs of ICP monitoring should utilize autoregulatory assessment to provide patient-specific thresholds for ICP and CPP. The use of non-invasive monitors of ICP is an attractive prospect but not yet supported by the technology.

## Conflict of Interest Statement

The authors declare that the research was conducted in the absence of any commercial or financial relationships that could be construed as a potential conflict of interest.
